# Tissue-Specific Immune Gene Expression in the Migratory Locust, *Locusta Migratoria*

**DOI:** 10.3390/insects6020368

**Published:** 2015-04-16

**Authors:** Tamara Pulpitel, Mathieu Pernice, Stephen J. Simpson, Fleur Ponton

**Affiliations:** 1 School of Biological Sciences, The University of Sydney, NSW 2006, Australia; E-Mails: stephen.simpson@sydney.edu.au (S.J.S.); fleur.ponton@sydney.edu.au (F.P.); 2 Plant Functional Biology and Climate Change Cluster, University of Technology Sydney, New South Wales 2007, Australia; E-Mail: mathieu.pernice@uts.edu.au; 3 The Charles Perkins Centre, The University of Sydney, NSW 2006, Australia

**Keywords:** insect immunity, gene expression, tissue-specificity, locust

## Abstract

The ability of hosts to respond to infection involves several complex immune recognition pathways. Broadly conserved pathogen-associated molecular patterns (PAMPs) allow individuals to target a range of invading microbes. Recently, studies on insect innate immunity have found evidence that a single pathogen can activate different immune pathways across species. In this study, expression changes in immune genes encoding peptidoglycan-recognition protein SA (PGRP-SA), gram-negative binding protein 1 (GNBP1) and prophenoloxidase (ProPO) were investigated in *Locusta migratoria*, following an immune challenge using injected lipopolysaccharide (LPS) solution from *Escherichia coli.* Since immune activation might also be tissue-specific, gene expression levels were followed across a range of tissue types. For PGRP-SA, expression increased in response to LPS within all seven of the tissue-types assayed and differed significantly between tissues. Expression of GNBP1 similarly varied across tissue types, yet showed no clear expression difference between LPS-injected and uninfected locusts. Increases in ProPO expression in response to LPS, however, could only be detected in the gut sections. This study has revealed tissue-specific immune response to add a new level of complexity to insect immune studies. In addition to variation in recognition pathways identified in previous works, tissue-specificity should be carefully considered in similar works.

## 1. Introduction

Insects rely on innate immune defence mechanisms to elicit responses against invading microbes. Microbial recognition enables hosts to recognise conserved stereotypical, rather than unique, microbial structures that are common to a wide spectrum of microorganisms. The best-known examples of such structures, called pathogen associated molecular patterns (PAMPs), include the lipopolysaccharides (LPS) of gram-negative bacteria and peptidoglycans (PGN) of gram-positive bacteria [1] that, along with other PAMPs, are recognised by pattern-recognition receptors [2]. Pattern-recognition receptors are highly conserved between insects and mammals [3], however they have been shown to activate different immune pathways depending on the animal species. In *Drosophila*, the peptidoglycan recognition protein SA (PGRP-SA) and gram-negative bacteria binding protein 1 (GNBP1) form a protein complex that recognises gram-positive PGNs [4,5] thereby activating the Toll pathway [6,7]. In beetles, it has been shown that cell wall components of both gram-positive and gram-negative bacteria are recognised by PGRP-SA and GNBP1, respectively, and induce both Toll and prophenoloxidase (ProPO) activation [8]. Such results suggest that Coleoptera and Diptera may have different AMP gene induction systems, revealing the importance of further investigations into the activation of such immune components, particularly from a comparative physiology viewpoint.

Activation of innate immunity has been classically measured in targeted tissues such as the haemolymph and fat body. Limited information exists on immune activation in other body parts, however, such knowledge may not only provide a better understanding of insect physiology in the context of infection, but also allow identification of tissue types that are potentially more informative for the interpretation of insect immunity (see for instance [9]). In the present study, we used reverse transcription quantitative polymerase chain reaction (RT-qPCR) to follow and compare the expression of three immune-related genes across seven tissues in saline- and LPS-injected locusts.

The migratory locust (*Locusta migratoria*, *Orthoptera*: *Acrididae*) is a species of great biological and global economic importance, estimated to impact millions of people around the world [10,11]. Because the control of locust populations includes the use of infectious disease agents [12], obtaining a better understanding of locust immunity is essential. We investigated the gene expression of two molecules involved in PAMPs recognition: PGRP-SA and GNBP1. Gene sequences coding for ProPO, an enzyme involved in the melanisation cascade of insects, and also triggered by microbe-derived molecules such as LPS, were also examined [13]. With the possibility that different immune pathways might be activated in both a pathogen- [14] and tissue-dependent [9] manner, our study aimed to highlight new challenges of immune response detection and quantification.

## 2. Experimental Section

### 2.1. Insect Cultures, Treatments and Tissue Collection 

Freshly moulted adult *L. migratoria* were reared to 14 d maturity under gregarious culture conditions as previously described [15]. Insects were fed daily with fresh wheat grass and wheat germ and provided with water *ad libitum*. Two treatment groups (*n* = 10) were generated from the same stock cage. Locusts from group 1 were injected with 100 μg lipopolysaccharides (LPS) from *Escherichia coli* (purified by phenol extraction; Sigma Aldrich, St. Louis, MO, USA) (equating to approximately 10^11^ bacteria) that was resuspended in 20 μL insect saline [16]. Control locusts from group 2 were injected with 20 μL of saline only. Injections were made into the thoracic haemocoel via the intersegmental membrane between the third and fourth terga using a 50-μL volume Hamilton micro-syringe and a 26-gauge bevelled needle. Following injections, locusts were returned to gregarious culture conditions for 7 h before being bled and dissected.

Haemolymph was obtained by piercing the arthrodial membrane at the base of the hind leg, using a sterile needle, and the expelled fluid collected using a micropipette. Each insect was bled to obtain the maximum volume of haemolymph possible per individual, with a minimum of 10 μL used for RNA extractions. Locusts from which less than 10 μL haemolymph was obtained were discarded as this had previously been demonstrated to be an insufficient amount for obtaining a high quality RNA yield (unpublished data [17]). All haemolymph samples were frozen directly at −80 °C without fixation. Bled locusts were immediately dissected to obtain the head, hind femur, fat body, and gut. These tissues, although often in contact with each other, were dissected as cleanly as possible to minimize cross contamination between tissues. Locust guts were separated at the gastric caecae and malpighian tubules to obtain the three gut sections: Foregut, midgut and hindgut. Each gut region was dissected open and washed in insect saline to remove contents. All tissues were immediately placed in *RNAlater* (Ambion, Austin, TX, USA) and held at 4 °C overnight before long-term storage at −80 °C.

### 2.2. RNA Isolation and Reverse Transcription Quantitative PCR

The present study conforms to the MIQE guidelines: Minimum information for publication of quantitative real-time PCR experiments [18]. In this section we indicate the essential information *sensu* [18] required to allow reliable interpretation of the corresponding RT-qPCR results.

RNA extractions were performed using a combination of Trizol-chloroform extraction procedures and the Qiagen *RNEasy* Plus Mini kit protocol for animal tissues as previously described [19,20]. Tissues were placed in 1 mL Trizol reagent (Invitrogen, Carlsbad, CA, USA) and homogenised using 5 mm stainless steel beads in a tissue lyser (Qiagen) for 40 s at 25 Hz. Following a 15-min incubation at room temperature, samples were centrifuged for 10 min at 4 °C (12,000 g) and the supernatant retained and washed in ¼ volume chloroform for phase separation. An additional step was added to the protocol to shear genomic DNA (gDNA) prior to the addition of chloroform. Here the RNA-containing supernatant was aspirated three times using a 26-gauge syringe (Livingstone). After a 3-min incubation period at room temperature, samples were centrifuged again for 20 min at 4 °C (12,000 g) and the upper phase transferred to gDNA eliminator columns (*RNEasy* Plus Mini Kit, Qiagen). From this step, the RNA extraction procedure followed the kit protocol until RNA elution. Samples were eluted twice: Once with RNAse free water (supplied with kit), and the second time with the eluate from the first elution to obtain a higher RNA concentration. To eliminate any remaining gDNA the purified RNA was treated with a DNAse DNA-free kit (Ambion), following the manufacturers protocol. Total RNA was quantified using a Nanodrop ND-1000 spectrophotometer (Nanodrop Technologies) and the samples stored at −80 °C.

Complementary DNA (cDNA) was synthesised following the manufacturers protocol from the SuperScript^®^ VILO kit (Invitrogen). Reactions used 250 ng total RNA in DEPC treated water, 5× VILO™ reaction mix and 10× SuperScript^®^ enzyme mix in a 20-μL final reaction volume. No template controls (NTC) and no reverse transcription (no-RT) controls were also run for each sample. Reverse transcription reaction conditions included an initial incubation period of 10 min at 25 °C, followed by 60 min at 42 °C. The reaction was terminated at 85 °C for 5 min. All cDNA samples and controls were stored at −20 °C.

Primers (Sigma Aldrich) were designed for immune genes based on sequences obtained from the Migratory Locust EST Database (LocustDB) [21]. Immune genes selected were from sequences coding for peptidoglycan recognition protein SA (PGRP-SA, LMC_003396), gram-negative bacteria binding protein 1 (GNBP1, LMC_001357) and the prophenoloxidase encoding gene (ProPO, LMC_001102). Reference genes used were Actin and Elongation factor 1a (EF1a), two genes that have previously been shown to be stably expressed in locusts [19]. The software Primer3 (v.0.4.0) [22] was used to design primers with parameters 18–22 bp in length, 59–61 °C Tm, 40%–60% GC content, a maximum self-complementarity of 5 bases and a maximum 3' self-complementarity of 2 base pairs. Product size ranges were between 80–150 bp. All other parameters were kept as default. Primer sequences, melting temperatures (Tm), accession numbers and amplicon lengths are shown in Table 1.

**Table 1:** Primer sequences for immune genes encoding peptidoglycan recognition protein SA (PGRP-SA), gram-negative bacteria binding protein 1 (GNBP1) and prophenoloxidase (ProPO) used in RT-qPCR gene expression assays. Reference genes (Actin and EF1a) were used to normalise relative expression. Amplicon length, melting temperature and accession numbers are shown.

Accession Number	Gene Name	Primer (F/R)	Primer Sequence	Amplicon Length (bp)	Tm (°C)
JF915527	PGRP-SA	F	AGGAGTTCATGGAGGTGCAG	87	64.3
		R	GCCAAGACGGTGGAGTACAT		63.9
JF915523	GNBP1	F	GGGAAGAGTTCAACCACCAA	83	63.9
		R	GCAAGCGTAGATTTCCAAGG		63.5
FJ771024.1	ProPO	F	TGTGCCTCATTGTCGTTGTT	137	64.3
		R	TACCTGGACGTGTGCTGAAG		64.0
KC118986	Actin	F	CTTTTCCCTGTTTGCCTTTG	104	63.4
		R	AAATCTGGCACCACACCTTC		63.9
AB583233	EF1a	F	CAGCCTGTGACGTTCCTGTA	112	64.0
		R	ATTGACATTGCGTTGTGGAA		64.0

Triplicate first strand cDNA aliquots for each sample served as templates for RT-qPCR using SYBR Green PCR Master Mix (Applied Biosystems, Foster City, CA, USA) and a LightCycler480 instrument (Roche Diagnostics). Amplification reactions were performed manually in 384-well optical plates (Perkin Elmer/Applied Biosystems Divisions) in 5 μL total volumes, which included 1 μL of cDNA template (diluted 1:20), 2.5 μL SYBR Green PCR Master mix (Applied Biosystems) and 100 nM each primer and ultra-pure water (Invitrogen).

Quantitative PCR was carried out under the following sequential conditions: Incubation at 50 °C for 2 min, 95 °C for 10 min, 45 cycles of 95 °C for 15 s and 60 °C for 1 min. Reverse transcription qPCR efficiency was determined for each gene and each treatment using the slope of a linear regression model [23]. Relative standard curves for the gene transcripts were generated with serial dilutions of cDNA (*i.e.*, 1/3, 1/9, 1/27, 1/81, 1/243, 1/486 and 1/972). Stock cDNA, used for the relative standard curves, constituted a pool of cDNA from the different treatments. The corresponding RT-qPCR efficiencies (E) were calculated according to the equation: E = (10^[−1/slope]^ − 1) × 100 [24]. Dissociation-curve analysis after 45 cycles of amplification revealed that all primer pairs amplified a single PCR product. All PCRs displayed a coefficient of correlation superior to 0.99. Efficiencies ranged between 98% and 120% and were shown to be reproducible between the different plates. Sample controls (NTC and no-RT) ensured that all samples were free from DNA contamination.

### 2.3. Statistical Analyses

Expression levels were determined as the number of amplification cycles needed to reach a fixed threshold in the exponential phase of the PCR reaction [25]. This number is referred to as the quantification cycle (Cq) value, the standard name for the threshold cycle or crossing point value according to the RDML guidelines [26]. These Cq values were transformed into quantities via the standard curve using PCR efficiencies according to Vandesompele *et al.* (2002) [27]. Target gene expression was normalised using the geometric mean of the reference genes [27]. Differences in expression levels between saline- and LPS-injected insects were tested using t-tests. Since the same individuals were used to measure the immune gene expression levels in the different tissue types, differences in expression levels of each gene between body parts for each treatment were tested using Friedman tests with individual number as a blocking variable. Because the test required equal samples, we only used individuals for which we had data for all body parts. All statistical analyses were performed using Systat 12 (Systat Software, Inc., San Jose, CA, USA).

## 3. Results and Discussion

**Figure 1:**
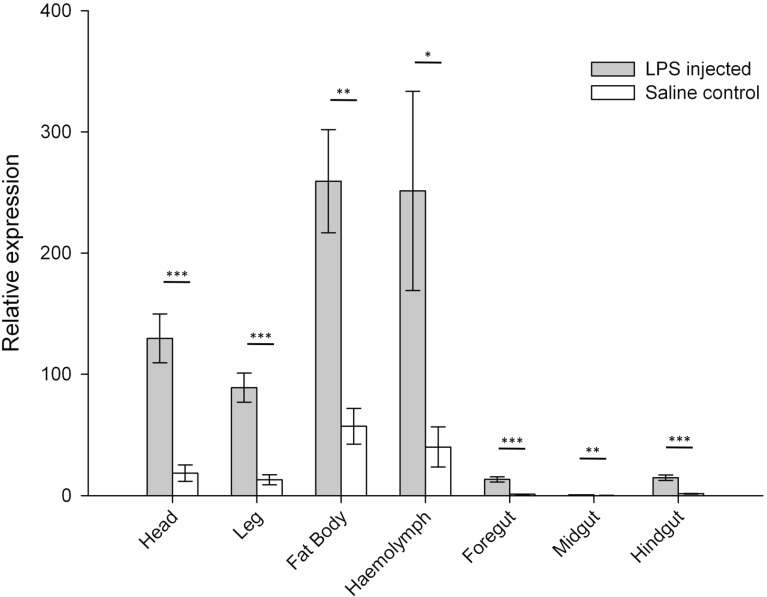
Relative expression levels of saline control and LPS-injected *Locusta migratoria* for the immune gene encoding peptidoglycan recognition protein SA (PGRP-SA) in seven tissue types. * *p* < 0.05; ** *p* < 0.01; *** *p* < 0.001. Error bars = ± SEM.

We compared gene expression levels for PGRP-SA, GNBP1 and ProPO between the seven selected body parts (*i.e.*, head, foregut, midgut, hindgut, haemolymph, fat body, leg) for LPS-challenged and control saline-injected *L. migratoria*. We observed increased expression in PGRP-SA in LPS-injected insects compared to saline controls in all seven tissues analysed (Figure 1). The magnitude of these increases differed depending on the body part being examined. The heads and legs of LPS-injected individuals showed the greatest rise in PGRP-SA expression, with 7.0- and 6.8-fold increases, respectively (Figure 1).

First reported in *Drosophila*, PGRP-SA has been described as a non-catalytic PGRP that activates the Toll pathway in response to the presence of most gram-positive bacteria [3]. However, investigations in other insects such as beetles have shown that PGRP-SA can also recognise gram-negative peptidoglycans, activating both Toll and Imd pathways [28]. Our results showed that injections of LPS from the gram-negative bacteria *E. coli* upregulated PGRP-SA expression in *L. migratoria*, as in beetles.

We also compared PGRP-SA expression between body parts for each treatment. As expected, different expression levels were found between the seven body parts within both infection treatments (control saline-injected insects: Friedman test, χ^2^ = 42.429, df = 6, *p* < 0.001; LPS-infected insects: Friedman test, χ^2^ = 47.810, df = 6, *p* < 0.001, Figure 1) indicating that tissue type plays a specific role in localised immune function. While greater expression levels were observed in the fat body and haemolymph for control saline-injected insects, PGRP-SA expression levels were greatest in the fat body and the head for LPS-injected locusts. Expression levels were lowest in all three of the gut regions for both LPS-injected insects and saline controls (Figure 1).

Wang *et al.* (2013) have recently measured PGRP-SA expression in the haemocytes, fat body and midgut of fungal-infected and uninfected *L. migratoria*, in both solitarious (solitary-reared) and gregarious (crowd-reared) phenotypes. In gregarious locusts, their results showed a higher relative expression of PGRP-SA in fat body than in haemocytes, with even lower (nearly undetectable) expression levels in the midgut [29]. Our results show a similar trend, however we were still able to detect some expression in midgut tissues despite expression levels being low relative to the other tissues. More specifically, expression levels of PGRP-SA were much lower in the midgut compared to foregut and hindgut. Buchon *et al.* (2013) have explored transcriptome variations in *Drosophila* gut regions, also showing that PGRP-SA expression differs between the different gut regions [30].

**Figure 2:**
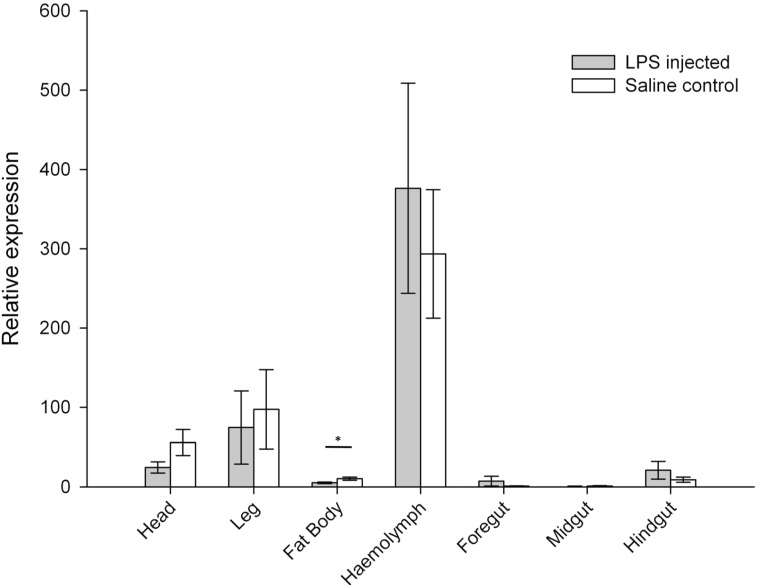
Relative expression levels of the gene coding for gram-negative binding protein 1 (GNBP1) across seven tissue types in LPS-injected and saline control *Locusta migratoria.* Error bars = ± SEM. * *p* < 0.05.

Expression of GNBP1 was not significantly upregulated in LPS-injected locusts (Figure 2), and was even downregulated in the fat body. Gram-negative bacteria binding proteins are a second class of immune recognition protein, initially purified from the haemolymph of the silkworm *Bombyx mori* and shown to have a strong affinity for the proteins at the surface of gram-negative bacteria [31]. In *Drosophila*, GNBP1 is implicated in immune response to gram-positive as well as gram-negative bacterial infection [32], and has been additionally shown physically to interact with PGRP-SA and to hydrolyse gram-positive peptidoglycan [4]. In locusts, GNBP1 seems to perform differently. Wang *et al.* (2013) compared GNBP1 expression levels in the fat body of locusts injected with different types of pathogen-associated molecular patterns [29]. These authors found an increase in GNBP1 gene expression in insects injected with laminarin (simulating fungal infection) and peptidoglycan but, similar to our results, found no upregulation of GNBP1 by LPS. From our study we can extend these observations to other tissues with no demonstration of enhanced GNBP1 expression in either fat body or any of the additional six tissue types examined (Figure 2). Gene expression levels however, were significantly different between tissue types for control saline-injected (Friedman test, χ^2^ = 42.696, df = 6, *p* < 0.001) and LPS-injected insects (Friedman test, χ^2^ = 42.381, df = 6, *p* < 0.001, Figure 2).

**Figure 3:**
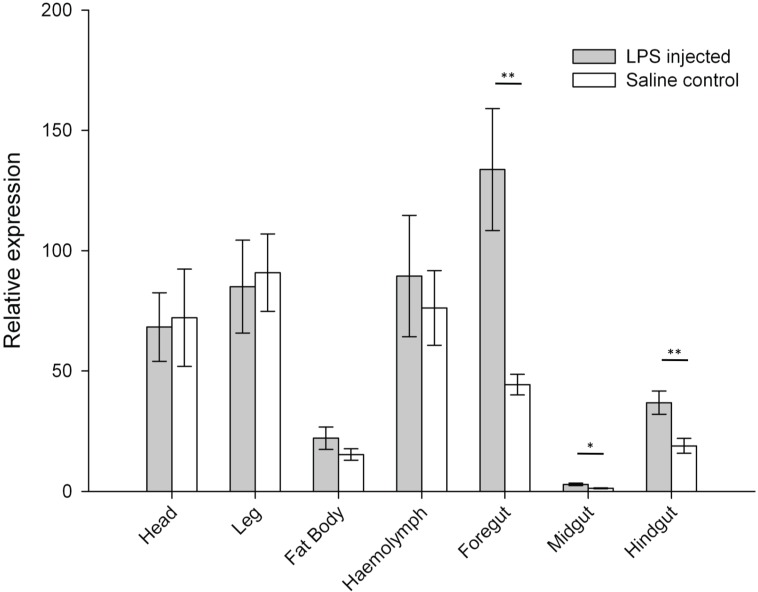
Relative expression levels of the gene coding for Prophenoloxidase (ProPO) across seven tissue types in LPS-injected and saline control *Locusta migratoria*. Error bars = ± SEM. * *p* < 0.05, ** *p* < 0.01.

We found a similar pattern of variation for both treatments, with expression levels being greater in the haemolymph, head and leg, and lower in the foregut and midgut (Figure 2). Expression levels of the gene coding for prophenoloxidase (ProPO) were significantly affected by the LPS immune challenge in all three gut segments, with increases in ProPO expression in LPS-challenged insects compared to saline controls (Figure 3). No effects of LPS-injection were detected in the other body parts (Figure 3).

Prophenoloxidase expression level was significantly different between body parts for insects injected with saline (Friedman test, χ^2^ = 40.500, df = 6, *p* < 0.001) and LPS (Friedman test, χ^2^ = 43.048, df = 6, *p* < 0.001), with lower expression levels in fat body and midgut compared to the other body parts for both treatments (Figure 3).

The prophenoloxidase–activating system is an important component in the humoral immune response of both insects and crustaceans [33,34]. This enzyme is an inactive zymogen of phenoloxidase, which is activated by Ca^2+^dependent proteolysis in response to microbial invasion [35]. Phenoloxidase catalyses several oxidation reactions by which melanin is synthesised through the release of reactive intermediate metabolites (e.g., quinones). Phenoloxidase-associated local melanisation, cuticular sclerotisation, wound healing and pathogen sequestering are all central defence responses of insects [34]. These responses are triggered by non-self recognition and frequently associated with clotting and physical encapsulation of invading parasites [36,37]. Phenoloxidases also serve multiple fundamental roles within insect developmental physiology [36]. Relatively little, however, is known about the molecular mechanisms that regulate ProPO production and activation. The production and regulation of precursor activation enzymes such as ProPO may be largely controlled and constitutively expressed by haemocytes in the haemolymph of insects [38]. Recent experiments in bees and mosquitoes, however, have found varying ProPO expression across different tissues including the integument, midgut and fat body, suggesting its synthesis in sites other than only haemocytes [39,40,41,42].

Cerenius and Söderhäll (2004) suggest that haemocyte-produced ProPO might infiltrate and accumulate in other tissues [36]. Haemocytes may therefore play a large role in ProPO synthesis in many tissues, which could explain why we found high ProPO expression across several different tissues in this study. Furthermore, although we observed differential ProPO expression across the seven different body parts, it was only upregulated in gut tissues following LPS-injection. This indicates that whilst ProPO is constitutively expressed in most tissues, the gut may be one of the first to respond to infection. Even though ProPO levels are not the highest in the gut of saline-injected controls, LPS injection triggered the greatest increase in ProPO gene expression. Since the gut region is a primary site of pathogen invasion, the observed increase in ProPO expression in response to LPS injection could reflect a role in the first line of defence to ingested pathogens.

The transcriptional response of ProPO in other insect studies appears to vary conditionally throughout the literature, revealing the importance of conducting further investigation into how this enzyme is regulated. Previous studies on the mosquito, *Anopheles gambiae*, found no expression increase of the ProPO gene in bacteria-challenged treatments, yet stimulation with 20E (an ecdysteroid hormone that regulates ecdysis) did induce ProPO upregulation [39,43]. Similarly no ProPO upregulation was found in the fall webworm *Hyphantria cunae* and in *Armigeres subalbatus* following infection compared to other immune-responsive genes (e.g., GNBP, defensin) [44,45]. In contrast, microfilarial worm injection into the blackfly, *Simulium damnosum*, has been shown to evoke upregulation of ProPO [46]. Knock-down experiments in a range of species have further demonstrated a reduced ability to fight infection, with a reduction in phagocytosis and nodule-formation as well as heightened susceptibility to infection by pathogens [42]. Along with the results from our study, understanding how ProPO is regulated across different species, tissues and in response to different immune challenges is complex. With recent evidence that PPO activation is also influenced by GNBP and PGRP [42], the importance of investigating the links between the expressions of such proteins in relation to ProPO is clear.

## 4. Conclusions

The results of our study indicate that three locust genes involved in pathogen recognition and immune activation show clear differences in their level of expression, not only across tissue types but also in response to injection with LPS. It is of particular interest that, following injection with LPS, gut-segments were the only tissues that showed increases in ProPO expression revealing a potentially newfound contribution of ProPO to the locust immune system. The results observed for this gene in particular emphasise the importance of tissue selection in conjunction with genes of interest and pathogen type when performing immune assays using RT-qPCR. In this instance, assays targeting more commonly used tissues, such as fat body and haemolymph, would not have revealed the same treatment response in *L. migratoria*. Alternatively, PGRP-SA showed a more general response to LPS, with significant increases in gene expression across all tissue types tested indicating that this gene may have a broader capacity to recognise and activate pathways to counter invading microbes. Perhaps counter-intuitively, GNBP1 showed a change in gene expression in response to LPS injection only in fat body, with a change that was opposite to that expected. The occurrence of tissue-specific immune response adds another level of complexity to the insect immune system. Consequently, when seeking to understand the general principals of immune action, and in the context of comparative locust biocontrol, such complexities should be taken into particular consideration.
